# Effect of methionine-35 oxidation on the aggregation of amyloid-β peptide

**DOI:** 10.1016/j.bbrep.2015.07.017

**Published:** 2015-07-30

**Authors:** Merlin Friedemann, Eneken Helk, Ann Tiiman, Kairit Zovo, Peep Palumaa, Vello Tõugu

**Affiliations:** Department of Gene Technology, Tallinn University of Technology, Akadeemia tee 15, 12618 Tallinn, Estonia

**Keywords:** Aβ, Alzheimer's amyloid peptide, AD, Alzheimer's disease, HFIP, 1,1,1,3,3,3-hexafluoro-2-propanol, OS, oxidative stress, ROS, reactive oxygen species, ThT, Thioflavin T, Alzheimer's disease, β-amyloid, Copper(II)ion, Methionine oxidation

## Abstract

Aggregation of Aβ peptides into amyloid plaques is considered to trigger the Alzheimer’s disease (AD), however the mechanism behind the AD onset has remained elusive. It is assumed that the insoluble Aβ aggregates enhance oxidative stress (OS) by generating free radicals with the assistance of bound copper ions. The aim of our study was to establish the role of Met35 residue in the oxidation and peptide aggregation processes. Met35 can be readily oxidized by H_2_O_2_. The fibrillization of Aβ with Met35 oxidized to sulfoxide was three times slower compared to that of the regular peptide. The fibrils of regular and oxidized peptides looked similar under transmission electron microscopy. The relatively small inhibitory effect of methionine oxidation on the fibrillization suggests that the possible variation in the Met oxidation state should not affect the in vivo plaque formation. The peptide oxidation pattern was more complex when copper ions were present: addition of one oxygen atom was still the fastest process, however, it was accompanied by multiple unspecific modifications of peptide residues. Addition of copper ions to the Aβ with oxidized Met35 in the presence of H_2_O_2_, resulted a similar pattern of nonspecific modifications, suggesting that the one-electron oxidation processes in the peptide molecule do not depend on the oxidation state of Met35 residue. Thus, it can be concluded that Met35 residue is not a part of the radical generating mechanism of Aβ–Cu(II) complex.

## Highlights

• Aβ peptides with oxidized Met35 residue fibrillize three times slower than the reduced peptide.
• Met35 is the only residue in Aβ peptide that is oxidized by H_2_O_2_ in the absent of copper ions.
• In the presence of copper ions as catalyst multiple unspecific oxidative processes occur in Aβ.
• Previous oxidation of Met35 does not affect the unspecific oxidation in the presence of copper ions.

## 1 Introduction

Alzheimer's disease (AD) is characterized by progressive neuronal death and the accumulation of protein aggregates in certain brain areas. The aggregation of amyloid-β (Aβ) peptide into extracellular amyloid plaques is identified as the key molecular event in AD, however, the molecular cascade leading to the death of neurons remains elusive. The increase of oxidative stress (OS) levels in certain brain areas is also characteristic to the disease and this increase is assumed to be an early event in AD [1], [2]. The major risk factor for the pathogenesis of AD is aging and according to the free radical hypothesis the major cause of aging is the accumulation of ROS and the accumulation of oxidative damage [3]. The relationship between OS and AD progression is a complex one: elevated levels of OS can be both, the risk factor and the consequence of AD progression [4], [5]. The toxicity of Aβ aggregates is assumed to arise from their ability to generate free radicals [6], [7], which depends on the presence of copper ions.

Considering the involvement of Aβ in oxidative processes, the Met35 is one of the most intriguing amino acid residues in the peptide molecule. Met35 has the most easily oxidized side chain in the peptide and it is partially oxidized in post mortem amyloid plaques [8], [9]. There has been many speculations about the role of Met35 in the formation of amyloid plaques and peptide toxicity: it has been suggested that Aβ mediated generation of ROS is initiated by the Met 35 residue [10]. On the other hand, a particular function in neuroprotection is also proposed for Met35 due to the antioxidant character of the thioether group [11], [12]. The suggestions about the involvement of Met35 in toxic mechanisms is supported by the observation that Met35 oxidation state is critical for Aβ synaptotoxicity – the oxidized form was clearly less toxic that the reduced one [13]. It has been shown that oxidation of Met35 reduces the Aβ42 toxicity in human neuroblastoma cells [14], [15], however, the substitution of Met35 to valine and norleucine had no effect on the toxicity of Aβ [16], [17].

Methionine residues can be oxidized by two different mechanisms [18]. First, they can be spontaneously oxidized to methionine sulfoxide in a two electron process by some oxidants such as H_2_O_2_ and molecular oxygen. This oxidation pathway can be considered relatively benign since no reactive oxygen species or free radicals are formed. The oxidation of methionine to sulfoxide is a common reaction in living organism and the oxidation can be reversed by the methionine sulfoxide reductases, enzymes that reduce the sulfoxide and are an essential part of redox detoxification mechanisms in the living organisms. Methionine oxidation to sulfoxide is demonstrated to inhibit fibril formation [12] and this observation has also lead to various speculations about the essential role of the oxidation of Met35 in AD. The sulfoxide can be further oxidized to sulfone, however, this reaction does not occur in physiological environments.

From the viewpoint of AD progression the second mechanism, one-electron oxidation of methionine to a highly reactive radical cation intermediate, is more intriguing. The single-electron mechanism can be catalyzed by copper ions that can have an essential role in AD etiology since they bind to Aβ with high affinity and enhance its aggregation whereas the electrochemically active copper ions buried within the aggregates can generate ROS and cause OS [19], [20]. An intriguing question is whether the radical cation generated from Met35 is an essential intermediate in the pathway of Aβ generated oxidative stress or is this just a supplementary process that does not contribute to ROS generation and OS. In order to answer these questions we studied the kinetics and products of the oxidation of Aβ by H_2_O_2_ in the presence and absence of copper ions as the catalysts of one electron oxidation processes. Methionine oxidation slightly decreased the rate of in vitro fibrillization of the Aβ peptides but did not changed its ability to catalyze the formation of ROS.

## 2 Materials and methods

### 2.1 Materials

Lyophilized Aβ40 and Aβ42 peptides (ultra pure, recombinant) NaOH salts or HFIP forms were purchased from rPeptide (Athens, USA). HEPES, Ultrapure, MB Grade was from USB Corporation (Cleveland, USA), 1,1,1,3,3,3-hexafluoro-2-propanol (HFIP) and Thioflavin T (ThT) were from Sigma Aldrich (St. Louis, USA). NaCl was extra pure from Scharlau (Barcelona, Spain). All solutions were prepared in fresh MilliQ water.

### 2.2 Sample preparation

Stock solution of Aβ peptides was prepared as follows: 1 mg of the peptide was dissolved in HFIP at a concentration 500 μM to disassemble preformed aggregates [21]. The solution was divided into aliquots, HFIP was evaporated in vacuum and the tubes with the peptide film were kept at −80 °C until used. Before using the Aβ HFIP film was dissolved in water containing 10 mM NaOH at a concentration of 10–20 μM. After 5 min incubation the Aβ stock solution was dissolved with buffer and used for experiments.

### 2.3 Fluorescence spectroscopy

Fluorescence spectra were collected on a Perkin-Elmer LS-45 fluorescence spectrophotometer equipped with a magnetic stirrer. Fibrillation was monitored using ThT fluorescence. If not otherwise stated, fresh Aβ stock solution was diluted in 20 mM HEPES and 100 mM NaCl, pH 7.4 containing 3.3 μM of ThT to a final concentration of 5 μM. 400 μl of each sample was incubated at 40 °C if not otherwise stated. ThT fluorescence was measured at 480 nm using excitation at 445 nm.

### 2.4 Data analysis and kinetics of fibril formation

The kinetics of Aβ fibrillation could be described as sigmoid curves and the aggregation parameters were determined by fitting the plot of fluorescence intensity versus time to Boltzmann curve

(1) $$y=\frac{A_{2}-A_{1}}{1+e^{-k(t-t_{0})}}+A_{2}$$

where *A*_1_ is the initial fluorescence level, *A*_2_ corresponds to the fluorescence at maximal fibrillation level, *t*_0_ is the time *t* when fluorescence is reached half maximum and *k* is the rate constant of fibril growth.

### 2.5 Transmission electron microscopy (TEM)

An aliquot of 5 μl of sample was loaded on a Formvar-coated, carbon-stabilized copper grid (300 mesh from Ted Pella Inc., Redding CA). After 1 min, the excess solution was drained off using a Whatman filter paper. The grid was briefly washed and negatively stained with 5 μl of 2% uranyl acetate. The grid was air-dried and then viewed on a Tecnai G2 BioTwin transmission electron microscope (FEI, Japan) operating with an accelerator voltage of 80 kV. Typical magnifications ranged from 20,000 to 60,000×.

### 2.6 Kinetics of Aβ oxidation

20 Aβ M of alkaline Aβ solution in 0.1% NaOH was diluted with equal volume of 0.1 M phosphate buffer containing hydrogen peroxide (H_2_O_2_) and Cu(II). Final concentrations of H_2_O_2_ was 1% and the concentrations of copper ions were 0.1 and 10 µM. The kinetics of Aβ oxidation and the disappearance of monomers from the solution were monitored with matrix-assisted laser desorption/ionization mass spectrometry (MALDI MS) using the energy absorbing matrix α-cyando-4-hydroxycinnamic acid (CHCA) [22]. Matrix CHCA was dissolved in 60% acetonitrile and 0.3% trifluoroacetic acid to a concentration of 10 mg/ml, containing 0.3 µM bovine insulin as an internal standard. Samples were mixed with matrix (1:3 ratio of sample: matrix) and 1 µl was spotted on the MALDI plate. MALDI MS spectra were acquired by *Voyager-DE™ STR Biospectrometry Workstation* in linear mode using automated program. Instrument parameter/settings: accelerating voltage 25,000 V; mass range (*m*/*z*) 1500–10,000 Da; delay time 485 ns; grid voltage 93%; laser intensity 2200 V, shots per spectrum – 40; accumulated spectrum – 10.

The site of oxidation was determined by sequencing the oxidized peptide on ESI-MS. 10 µM Ab42 in 100 mM ammonium acetate buffer (pH 7.5) was injected into the electrospray ion source of QATAR Elite ESI-Q-TOF MS instrument (Applied Biosystems) by a syringe pump at 7 µl/min. ESI MS spectra were recorded for 5 min in the *m*/*z* region from 300 to 1500 Da with the following instrument parameters: ion spray voltage 5500 V; source gas 45 l/min; curtain gas 20 l/min; declustering potential 45 V; focusing potential 260 V; detector voltage 2450 V; collision energy 50 V; collision gas 5 l/min; precursor-ion mode.

Sodium dodecyl sulfate polyacrylamide gel electrophoresis (SDS-PAGE) was performed using *Mini-PROTEAN Tetra System* (Bio-Rad). Samples were mixed with loading buffer (0.36 M Bistris, 0.053 M Bicine, 15% glycerol, 1% SDS, 0.004% bromophenol blue), maintained at room temperature, applied to Bicine–Tris 15%T/5%C gel and resolved in a cathode buffer with 0.25% SDS (110 V) [23]. Gels were fixated in glutaraldehyde/borate buffer solution for 45 minutes and stained with silver according to a protocol [24].

## 3 Results

### 3.1 Oxidation of Met35 by H_2_O_2_

**Fig. 1:**
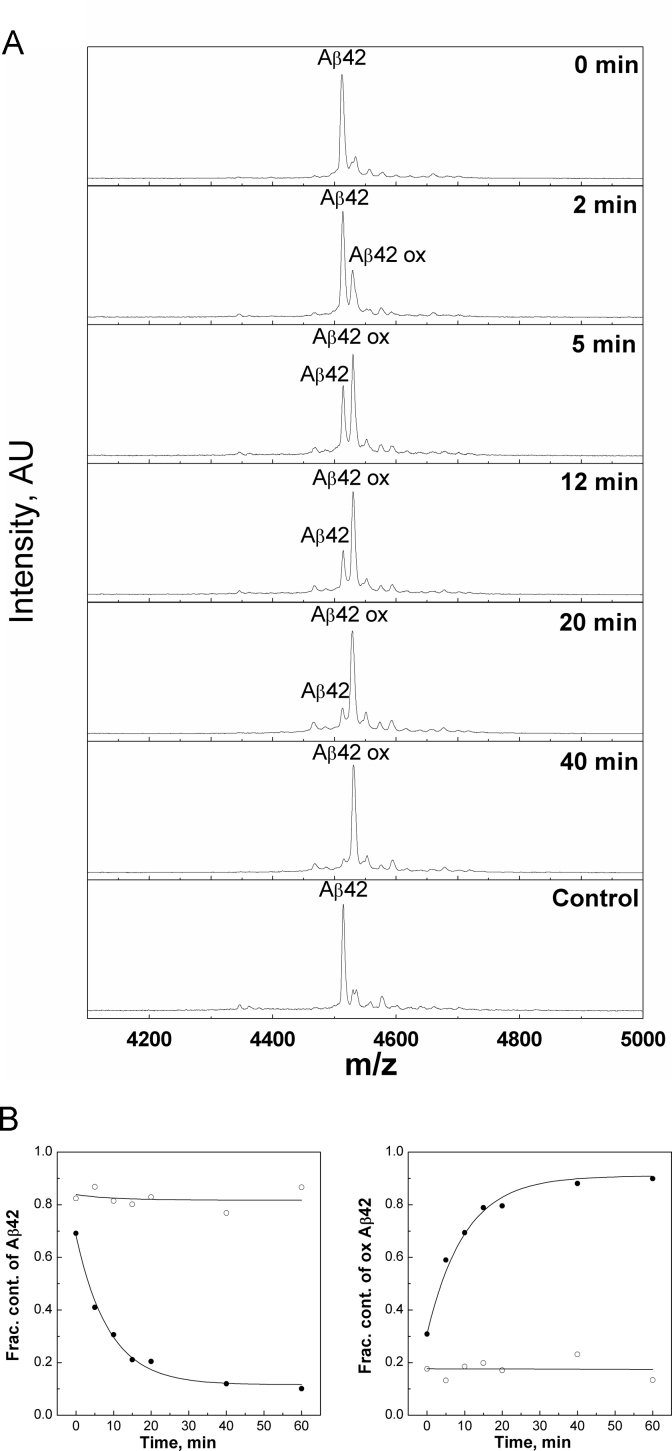
Oxidation of Aβ42 peptide with H_2_0_2_ in the absence of copper ions. (A) MALDI MS spectra of Aβ42 incubated in 20 mM HEPES at pH 7.3 in the presence of 1% H_2_0_2_. Samples were taken at time intervals shown in the legend; (B) kinetics of oxidation, ● − 1% H_2_0_2_, Ο-control; *k*=(0.114± 0.012) min^−1^.

The oxidation of Aβ42 by H_2_O_2_ was studied by MALDI mass spectrometry. Fig. 1 shows that the peptide was easily oxidized in the presence of 200 mM H_2_O_2_. During the incubation with hydrogen peroxide the molecule mass of the peptide increased by 16 units and the process was almost complete within 40 min. The oxidation of Met35 residue was confirmed by ESI MS/MS sequencing of the resulting oxidized peptide (Supplementary Fig. 1). Kinetic analysis of the MALDI MS data (Fig. 1B) showed that the disappearance of reduced Aβ and the increase of AβMet35ox followed the first order kinetics. In the absence of copper ions AβMet35ox was the only product of the oxidation: no side products were detected in a significant amount.

**Fig. 2:**
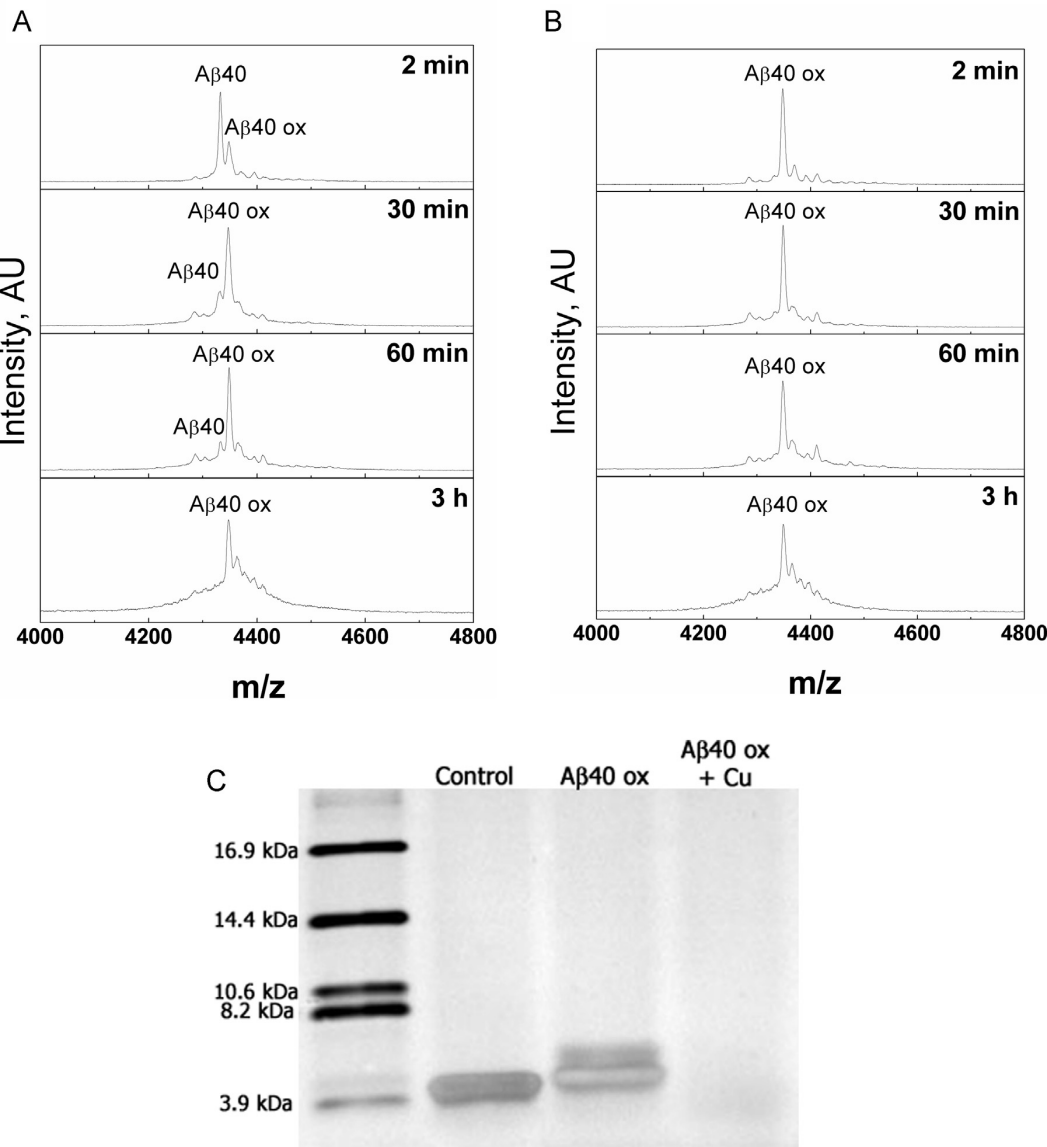
Oxidation of Aβ40 peptide with H_2_0_2_ in the presence of copper ions. MALDI MS spectra of Aβ42 incubated in the presence of 1% H_2_0_2_ at pH 7.3 in 20 mM HEPES. Samples were taken at time intervals shown in the legend; A – native peptide; B – peptide was oxidized with H_2_0_2_ for 40 min before adding copper ions. C-SDS Page of Aβ40: Control, untreated peptide, Aβox, oxidized with H_2_0_2_ in the absence of copper ions and Aβ ox+Cu refers to oxidized peptide treated with H_2_0_2_ in the presence of copper ions for 3 h.

The oxidation pattern of Aβ in the presence of copper ions was more complex. As the copper complex of Aβ42 tends to form a precipitate during 30 min of incubation [25] the oxidation in the presence of copper ions was studied using Aβ40 peptide. Fig. 2 shows that the addition of a single oxygen atom was still the fastest process; however, the widening of the peptide peak in MS showed that subsequently a large number of non-specific modifications (oxygen additions and elimination) reactions in the Aβ molecule occurred. When Cu(II) ions were added to the reaction mixture after the oxidation of Met35 by H_2_O_2_ (Fig. 2B), a similar pattern of nonspecific modifications was observed, suggesting that the single-electron oxidation processes in Aβ does not depend on the oxidation state of Met35. In principle, a peptide dimer can also form by dityrosine crosslinking during peptide oxidation. SDS PAGE (Fig. 2C for Aβ 40 and Supplementary Fig. 2 for Aβ42) shows that peptide dimers do not form under our experimental conditions, peaks of dimers and trimers were also not detected by MALDI MS.

### 3.2 Fibrillization of AβMet35ox

**Fig. 3:**
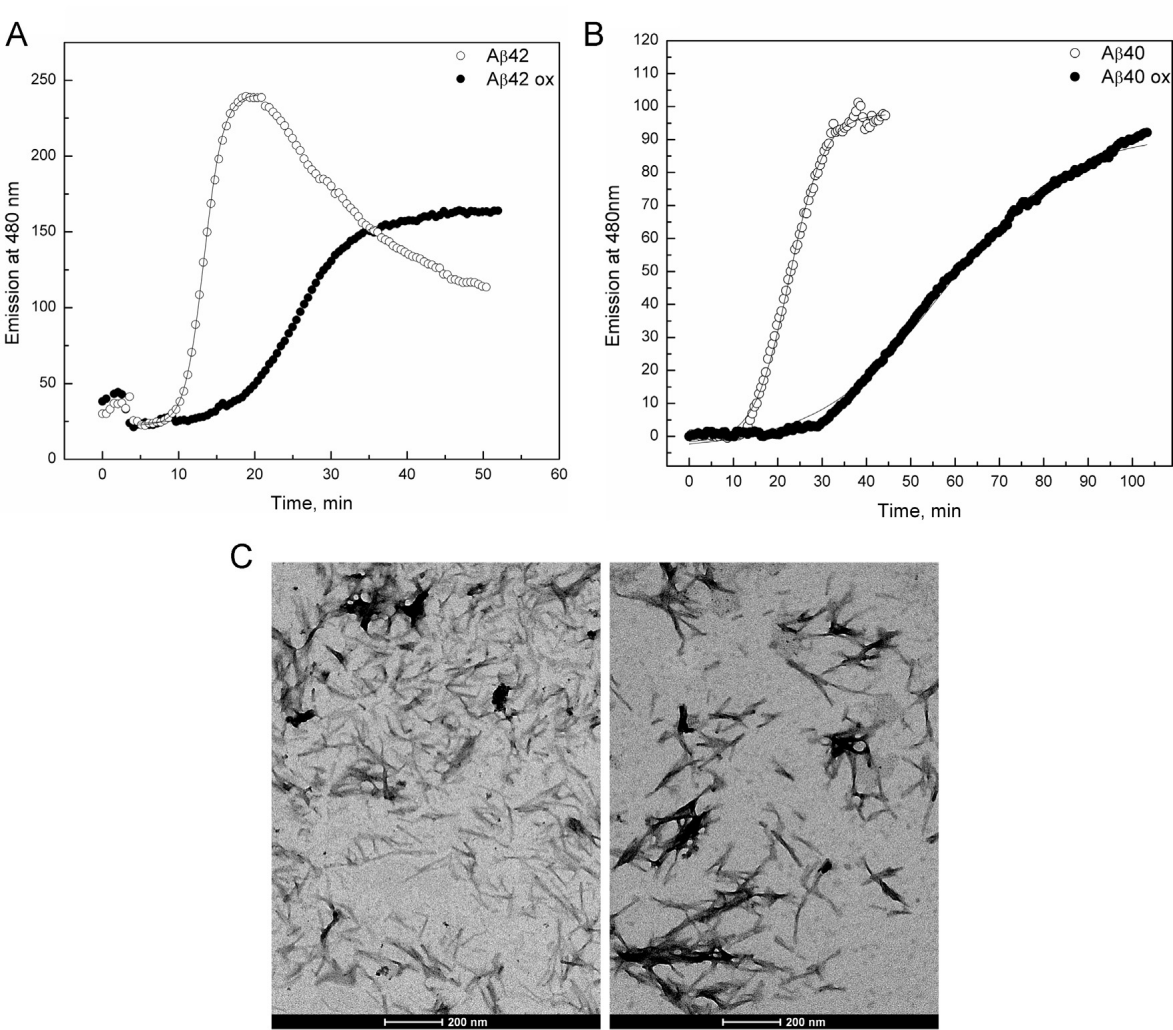
Fibrillization of Aβ peptides with reduced and oxidized Met35 residues at pH 7.3, 20 mM HEPES, 100 mM NaCl, 5 μM ThT. A – Fibrillization of Aβ42: 4 μM Aβ42 37 °C: Curves correspond to *k*=(1.30±0.02) min^−1^, *t*_lag_=11.8 min^−1^ for reduced and *k*=(3.83±0.05) min^−1^, *t*_lag_=24.9 min^−1^ for Met35ox peptide. B-Fibrillization of Aβ40: 5 μM Aβ40; 50 °C; Curves correspond to *k*=(3.92±0.09) min^−1^, *t*_lag_=22.6 min^−1^ for reduced peptide and *k*=(14.1±0.3) min^−1^, *t*_lag_=14.0 for the oxidized peptide; C-TEM images of Aβ40 fibrils, left Aβ40ox; right – Aβ40 control.

The fibrillization of Aβ was studied under the conditions of intensive agitation where the fast fibrillization of the peptide proceeds with good reproducibility [25], [26]. Incubation of unpurified oxidized peptide under these conditions did not reveal an increase in ThT fluorescence; however, the peptide peak in the MALDI MS spectrum disappeared showing fibrillation [22] and in a TEM image typical non-matured fibrils were observed. As the oxidizing agent can interfere with the ThT based detection method we repeated the experiments after removing the oxidizing solution by lyophilization and redissolving the peptide in HEPES buffer. Both oxidized peptides, Aβ42 and Aβ40, showed a typical sigmoidal fibrillization curve (Fig. 3A and B), whereas the value of the fibrillization rate constant, *k*, was threefold lower than that of the regular peptide. The lag-phase for the oxidized peptide was also longer however, the resulting fibrils look similar in TEM (Fig. 3C).

## 4 Discussion

In this work we studied the oxidation of Aβ in the presence of two physiologically relevant redox-active compounds H_2_O_2_ and copper ions. Low concentrations of H_2_O_2_ are always present in living organisms and take part in various redox processes; copper ions bind to Aβ with high affinity in a catalytically active form and are present in amyloid plaques. The aim of the study was to establish the role of the Met35 residue in the oxidation and peptide aggregation processes. In the absence of copper ions the Met35 residue in Aβ molecule was readily oxidized to sulfoxide (Fig. 1 and Supplementary Fig. 1) and this was almost the only modification observed. Met35 oxidation only slightly inhibited Aβ fibrillization, thus, we suggest that the possible variation in the Met oxidation state should not have a large effect on the plaque formation in vivo. The amyloid fibril formation is a complex autocatalytic process and the fibrillization in agitated solutions models the fibril growth phase, but not the initiation of the fibrillization process or the formation of the protofibrils and first fibrils.

In the presence of copper ions that form a high affinity complex with Aβ, the oxidation was more complex, the addition of the first oxygen was still the fastest process, however, it was accompanied by unspecific modifications of several residues in the peptide. Met35 oxidation had no effect on the oxidative behavior of Aβ complex with copper ions – when Met35 was oxidized in the absence of copper, the addition of copper still lead to the appearance of diverse spectrum of oxidized peptides. These modifications may include the oxidation of His and Phe residues in Aβ and Glu 1 decarboxylation observed in the presence of oxygen and ascorbic acid [27]. Thus, it can be concluded that Met35 residue is not a part of the radical generating mechanism of the Aβ–Cu(II) complex.

The oxidation state of Met35 enhances the toxicity of the artificial truncated Aβ25-35, which affects mitochondria [28]. However, the high toxicity of this derivative is dependent on the C-terminal position of methionine and does not apply to the longer Aβ variants such as 25–36 [29], thus, this process is not related to the toxic effect of the full-length Aβ and amyloid plaques. It should also be noted that we did not observed the formation of potentially highly toxic Aβ dimers under our experimental conditions e. g. the oxidative dimerization of Aβ due to tyrosine crosslinking did not occur at considerably higher concentrations of the peptide and H_2_O_2_ than those in the brain. However, the oxidative dimerization of Aβ in vivo can be catalyzed by enzymes [30]. It should also be noted that the Aβ “dimers” from biological material have never been analyzed chemically e. g. they are not necessarily dimers, but they can be longer peptides containing the Aβ sequence [31].

Methionine added to the environment also does not serve as a reducing agent for the Cu(II)–Aβ complex [32], thus, it can be concluded that from the viewpoint of redox ability and fibril formation the possible oxidation of Met35 residue is not an important property of the peptide. However, Met35 can play a role in AD pathogenesis due to putative interactions in the biological systems. It has been shown that in a *Caenorhabditis elegans* model of inclusion body myositis the knockout of MSRA-1 reduces the aggregation of Aβ into insoluble aggregates [33], however in this case the aggregation is intracellular. Even small differences in the peptide aggregation properties may be crucial in triggering the molecular events leading to the disease, however the lower amyloidogenity and the unaffected ability to catalyze redox reactions when bound to copper ions suggest that Met35 oxidation is most likely not essential in AD. Recently it has been shown that Aβ with oxidized Met 35 that does not cross the neuronal plasma membrane and is not uploaded from the extracellular space has no effect on synaptic plasticity when applied extracellularly [34].
