# Supplementary material for: Effect of methionine-35 oxidation on the aggregation of amyloid-β peptide
Source: Biochem Biophys Rep. 2015 Jul 30;3:94–9. doi: 10.1016/j.bbrep.2015.07.017 (PMC5668694; doi:10.1016/j.bbrep.2015.07.017)
Supplement: Supplementary file 1 — Supplementary material [file mmc1.docx]

Supplementary Figure 1


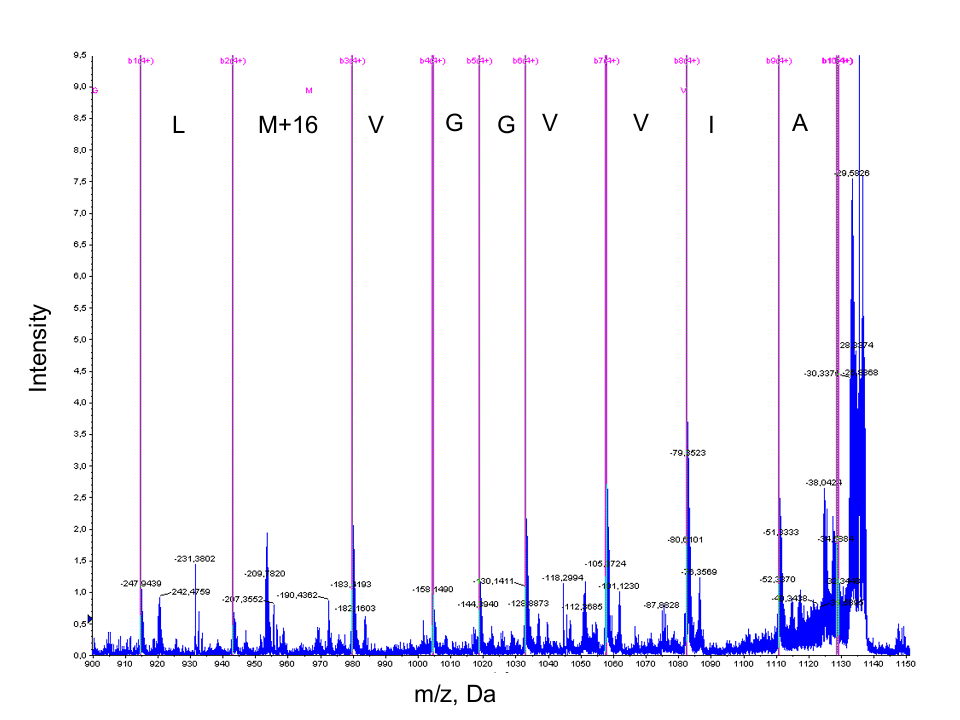
 ESI-MS-MS spectrum of oxidized Aβ 42. Mass of Met residue is increased by 16 units showing the oxidation of Met35.

Supplementary Figure 2.


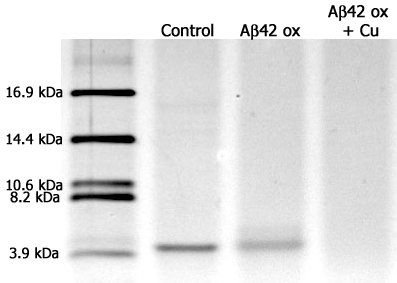


SDS Page of Aβ42: Control, untreated peptide, Aβox, oxidized with H_2_0_2_ in the absence of copper ions and Aβ ox + Cu refers to oxidized peptide treated with H_2_0_2_ in the presence of copper ions for 3 hour.
